# Patterns of smoking behavior among physicians in Yerevan, Armenia

**DOI:** 10.1186/1471-2458-6-139

**Published:** 2006-05-23

**Authors:** Paul C Perrin, Ray M Merrill, Gordon B Lindsay

**Affiliations:** 1 Department of Health Science, College of Health and Human Performance, Brigham Young University, Provo, Utah, USA

## Abstract

**Background:**

Physicians can play an important role in smoking prevention and control. This study will identify smoking prevalence among physicians in Yerevan, Armenia. It will also explore how the smoking behaviors of physicians, their perceived ability to influence patient smoking behavior, and their knowledge about health outcomes related to smoking influence their interaction with patients.

**Methods:**

A cross-sectional, self-administered, anonymous survey was conducted in July, 2004, among 12 healthcare facilities in Yerevan. Analyses are based on responses from 240 physicians, representing a 70% response rate.

**Results:**

The percentage of current smokers was significantly higher in men than women (48.5% vs. 12.8% regular and 6.8% vs. 4.5% occasional). Among current smokers, 52.7% of men compared with 13.0% of women had previously smoked in the presence of patients. Only 35.3% felt well prepared to assist patients to quit smoking. Physicians who smoke are less likely to ask their patients about their smoking behavior or believe their example is likely to influence their patients. Level of perceived preparedness to assist patients to quit smoking was positively associated with knowledge about known health risks associated with smoking.

**Conclusion:**

Smoking prevalence is high among physicians in the 12 healthcare facilities in Yerevan, and a large percentage of physician smoke in the presence of their patients. Physician smoking behavior and knowledge of smoking related health outcomes in Yerevan influences whether they counsel patients regarding smoking.

## Background

The global burden of disease resulting from tobacco smoking is well understood [1,2]. Tobacco smoking prevention and cessation efforts, primarily in developed countries, have been effective at reducing heart disease, cancer, and other smoking-related illnesses. Prevention efforts in the United States have combined elements of health education, cessation techniques, and legislative activity to address the smoking problem [3]. Large decreases in smoking prevalence illustrate the success of these efforts. In 1965, the percentage of current smokers 18 years of age and older was 51.9 for men, 33.9 for women; 42.1 for whites, 45.8 for blacks; and 42.4 overall [4]. By 2002, corresponding percentages were 25.2 for men (51% decrease), 20.0 for women (41% decrease); 23.6 for whites (44% decrease), 22.4 for blacks (51% decrease); and 22.5 overall (47% decrease) [4].

Contrary to favorable trends in smoking prevalence in the United States and elsewhere, patterns in smoking prevalence are less favorable in many developing countries. For example, in 2002 the World Health Organization (WHO) estimated that among adults in the Republic of Armenia 64% of males and 1.0% of females currently smoked [5]. In Yerevan, the capital of Armenia, a 2005 survey estimated smoking prevalence among people 16 years of age and older as 54.7% in males and 4.3% in females [6]. For all other provinces in Armenia, 61.6% of males and 1.1% of females were current smokers. These estimates place male smoking prevalence in Armenia among the highest in the world.

In recent years, cigarette consumption in Armenia has also increased, both overall and per-capita. Armenians smoked 5,800 million cigarettes in 1999, which represents a 9% increase over the 5,305 million cigarettes smoked in 1993 [7]. This cannot be solely attributed to population increase since annual per-capita cigarette consumption among those 15 years of age and older increased by 5% in Armenia during the 1990s, from 105 packs in 1993 to 110 packs in 1999.

The health implications of high smoking prevalence in Armenia are now being manifest in higher morbidity rates of heart disease, cancer, and respiratory diseases in the resident population [8]. In 1994, 94% of all lung cancer deaths in Armenia for males aged 35 to 69 were due to smoking [9]. An estimated 4,400 deaths in the country in 1995 may be attributed to tobacco smoking, among which 90% of those deaths were men. This represents about 26% of all male deaths in the country [9]. This is a significant increase compared with only a decade earlier, 1985, when 19% of male deaths were attributed to tobacco smoking [10].

Studies have shown that physicians and other health-care workers can act as important figures in reducing societal smoking prevalence [11-14], and can contribute to stemming the projected increase in mortality and morbidity from cigarette-related diseases [1,2]. The WHO recently reported that prevalence of current smokers among doctors in Armenia in the late 1990s was 80.6% of males and 42.0% of females [15]. The report did not address physician attitudes toward smoking and potential obstacles they face in smoking prevention and control efforts. The purpose of this study is to identify the smoking prevalence of physicians in Yerevan, Armenia. This study will also address the attitudes of physicians in Armenia toward smoking, and potential obstacles in tobacco prevention and control efforts. Related studies assessing the smoking behaviors and beliefs of healthcare personnel have previously been conducted in Bosnia-Herzegovina, Estonia, Finland, France, Ireland, Italy, Senegal, Spain, and the United States [16-26].

## Methods

### Study population

Armenia is a highly centralized population with over a third of its 3.3 million citizens living in Yerevan, which is the capital of the country. Armenia's healthcare system is a remnant of the socialized Soviet system. There are a comparatively high number of physicians in relation to the population. In 2003, there were 11,728 (36.5 per 10,000) physicians of all specialties in Armenia [8].

The study focused on the capital city, Yerevan, which has a population of approximately 1.2 million people. A study by the National Statistical Service of the Republic of Armenia published the smoking rates by region, showing the smoking rates in Yerevan to be approximately equal to the nation-wide smoking rates [27].

### Study design

A cross-sectional survey was conducted in July 2004. Criteria for inclusion in the study were practicing physicians with clinical interaction with patients and currently practicing at a licensed medical facility located in Yerevan. Health researchers and pharmacists were not included in the study.

Approval for the current study was granted by the Brigham Young University Internal Review Board prior to administering the survey.

### Instrument validity and reliability

The questionnaire used items from an instrument developed by the WHO and the International Union against Tuberculosis and Lung Diseases, specifically made for healthcare workers. This instrument was selected because of its use in the peer-reviewed literature [16,20] and a book [28]. Although the validity of the instrument has been established in other settings, we assumed it was applicable in Armenia. In addition, the constructs and variables it measures are consistent with the intent of the present study. A few questions were added to ensure the study objectives were met. The questionnaire was translated in-country by a professional translator. It was pre-tested for face validity among a group of 20 Armenian physicians, and minor changes in wording and format were made based on their recommendations.

### Data collection

A list of licensed medical facilities (i.e. hospitals and clinics) in Yerevan was compiled. Twelve facilities were randomly selected out of 36, and the survey was given to the administrators of these facilities. All of the selected hospital administrators agreed to participate in the study. Anonymous, self-administered questionnaires were distributed to the medical facilities. The number of questionnaires given to each medical facility reflected the number of physicians in each hospital. The hospital administrators then asked their physicians to complete and return the questionnaire. The completed questionnaires were then collected by the lead author.

In total, 400 questionnaires were distributed to 12 facilities, of which 313 were returned (78% response rate), and 280 were usable (70%). The skip patterns and answer codes on both the section assessing behavior and the section assessing attitudes were used incorrectly or not at all in the 33 questionnaires not used. Forty surveys were deleted because they were completed by nurses, laboratory personnel, or had missing information on their specialty. This left 240 completed questionnaires available for analyses.

### Data analysis

Frequency distributions were used to describe the data. Bivariate analyses were used to measure associations between selected variables, with statistical significance based on the chi-square (χ^2^) test for independence [29]. In addition, adjusted odds ratios and 95% confidence intervals were estimated using logistic regression [30]. Two-sided tests of significance were based on the 0.05 level against a null hypothesis of no association, unless otherwise indicated. Analyses were performed using SAS version 9.0 (SAS Institute Inc., Cary, NC, USA, 2003). Procedure statements used in SAS for assessing the data were PROC FREQ, PROC UNIVARIATE, and PROC LOGISTIC.

## Results

A description of the study participants according to selected demographic, behavioral, and policy variables are presented in Table 1. The mean age of participants was 43.5 years (SD = 12.0), with ages ranging from 22 to 75. The most frequent levels within each variable were ages 50–59, women, never smokers, workers in facilities banning smoking altogether, workers in facilities where smoking policies were enforced, and practitioners who felt only somewhat prepared to assist patients to quit smoking.

Bivariate analysis showed that smoking prevalence among female physicians in Armenia was significantly lower than among their male counterparts (Table 2). However, the percentage of female physicians who are current smokers is high. Of males with a history of smoking 26.0% are former smokers. Of females with a history of smoking 17.9% are former smokers.

A description of current physician smokers according to sex is presented in Table 3. This table shows that the majority of women started smoking after they reached 25 years of age, whereas the majority of the men started smoking at younger ages. Quit attempts did not significantly differ between men and women. Men were much more likely than women to have smoked in front of patients, but there was not a significant difference in the number of men and women who regularly smoke in front of patients. Finally, women were much more likely to indicate a readiness to quit within the next six months, albeit not significantly so.

Physicians were asked their level of agreement with nine statements about their perceived role in interacting with patients in the context of smoking (Table 4). The highest levels of agreement were with statements that health professionals should routinely advise patients who smoke to not smoke in front of children, to quit smoking if they smoke, and that they should regularly ask patients if they smoke. Physicians also had a high level of agreement with the statement that health professionals should not smoke in front of patients and that they should be a good example to their patients. The highest level of being unsure was with the statement that chances of a patient quitting increases when the doctor tells them to. The lowest levels of agreement were with the statements that health professionals should get training on cessation techniques and that health professionals who smoke are less likely to advise patients to stop smoking.

Physicians' smoking behavior may influence how and whether they counsel their patients regarding smoking. The questions for which smoking status influenced their responses to the opinion questions at the 0.1 level of significance are given in Table 5. Consistently, less than one-third of those agreeing with any of these statements were smokers. Disagreeing with these statements was consistently associated with a higher chance of being a current smoker.

There was no statistical association between level of preparedness to counsel a patient about their smoking and age and sex. A description of the association between level of preparedness to counsel a patient about their smoking and selected statements about smoking knowledge is presented in Table 6. Those who agreed with these true statements about smoking were more likely to feel well prepared to counsel patients. Those who were unsure or disagree with these true statements about smoking were more likely to feel unprepared to counsel patients.

## Discussion

Healthcare workers have been shown to play an important role in tobacco prevention [31-33]. Primary care physicians in particular are one of the most powerful groups at lowering the acceptability of smoking in various social contexts, a process often called "denormalization" [34]. The current study provides information that may be useful in designing smoking prevention and cessation programs that involve physicians in Armenia.

The results of this study demonstrate that many physicians in Armenia, rather than acting as the important resource they could be, may in fact be eroding the effect of tobacco prevention and control efforts by reinforcing the normalization of tobacco through their attitudes and practices. Approximately 55.3% of male physicians were current smokers and 52.7% have smoked in the presence of patients. As for female physicians, 17.3% were current smokers and 13.0% have smoked in the presence of patients. Those who have smoked in the presence of patients may believe that their behavior does not influence others, or that they do not fully understand the harmful effects of smoking. Recall that those who disagreed with statements: "healthcare workers are examples for their patients and the public," "healthcare workers should set a good example by not smoking," and "healthcare workers should regularly ask their patients about their smoking habits," were more likely to be smokers.

Cultural acceptance of smoking in Armenia may help explain the high smoking prevalence among physicians. The current study found smoking prevalence among physicians in Yerevan to be 55.3% in males and 17.3% in females. These results are lower than the WHO recently reported for physicians in Armenia – 80.6% in males and 42% in females [15]. Compared with the general adult male population in Yerevan, smoking prevalence among physicians is similar [6]. On the other hand, smoking prevalence is much higher among female physicians compared with the general female population in Yerevan (17.3% vs. 4.3%) [6]. Furthermore, smoking prevalence among women in Yerevan is approximately four times greater than that in the other provinces of Armenia [5,6]. Higher smoking prevalence among female physicians may reflect an attempt to gain greater affluence and liberation from old rural culture. It may also be that tobacco companies are more aggressively targeting women with higher socioeconomic status. It has been shown that among women in India cigarette smoking exists primarily among the urban elite classes of large cosmopolitan cities [35].

Studies have shown that physicians have often been at the forefront at quitting smoking [36,37]. An older study showed that California physicians who currently smoked decreased from 53% in 1950 to 10% in 1980 [38]. In comparison, the decrease in American men who smoked cigarettes was from 53% to 38% during the same time period. Two US studies conducted in the 1990s found that physicians displayed considerably lower smoking prevalence than the general population [39,40]. These studies identified smoking prevalence among physicians at 3%-4%, which is consistent with physicians having healthier lifestyle behaviors than the general population [41].

Healthcare professionals can help patients stop smoking by ensuring that counseling and pharmacological therapy is available [42], and actually counseling them about quitting [33]. A study involving the Women Physicians' Health Study in 1993 found that practicing a specific health habit (e.g., not smoking) significantly increased the likelihood of counseling patients about that habit [41,43]. Patients also find physicians more believable and motivating if the physician discloses their own positive health practice [41].

Only a minority of Armenian physicians felt well prepared to counsel patients to quit smoking. Those who did feel well prepared to assist patients to quit smoking were more likely to agree with true statements about smoking; those who felt unprepared to assist patients to quit smoking were less likely to agree with true statements about smoking. Hence, perceived preparedness to assist patients to quit smoking is associated with knowledge about adverse health outcomes previously linked to smoking.

Physicians who do not smoke are more likely than those who do to provide advice to quit [43]. Nurses can also have an impact on lowering smoking among patients [32]. If only half of all nurses worldwide helped one patient per month quit smoking, more than 12 million smokers would overcome their addictions every year [14]. Evaluation of the smoking behaviors of nurses and their tendency toward counseling patients in Armenia is an area for further study.

Social policies aimed at controlling cigarette smoking can also have a significant impact on smoking rates [44]. Legislation passed by Armenia's parliament and adopted in January 2005 increased fines and outlawed smoking in schools, on public transportation, and in other public places. Smoking was also banned for teenagers under 16. The legislation further prohibited smoking in cultural institutions and at sporting events. Tobacco products without warnings on the dangers of smoking were destroyed. Healthcare workers can have a leadership role to play in supporting such policies.

Physicians in Armenia may not be taking full advantage of windows of opportunity to identify smokers and provide smoking advice. The fact that a minority of physicians indicated that they felt well prepared to assist a patient with a smoking problem, in conjunction with the fact that a majority of male physicians are current smokers and that the smoking prevalence of female physicians is over seven times that of the national prevalence, indicates a need for more developed medical system intervention.

Research is warranted in Armenia to determine the feasibility of smoking prevention interventions. A study targeting the patients of healthcare workers, as well as the population as a whole, would help us understand the potential impact such interventions could have in Armenian society. Such studies could measure the percentage of the population who visit healthcare facilities, how often they do so, and the extent a physician's advice influences patient decisions. Studies could also ascertain whether the physicians would use smoking cessation materials if they were provided, and whether physicians are actively involved in promoting health policy related to smoking prevention and control.

Because of the hierarchical administrative structure of the hospital, it was impossible to obtain direct access to the physicians under study. Therefore, we relied on the hospital administrators to distribute the questionnaire. This could have caused selection bias. This limitation was overcome in part by distributing the same number of surveys to a facility as the number of eligible physicians therein.

Another potential source of bias arises from the response rate. Some of the questionnaires were not distributed to physicians (in a few cases hospital administrators admitted they were unable to distribute all of the questionnaires because some workers were on vacation). In addition, it is possible not everyone filled out the questionnaire once it was received. The completed questionnaire retrieval rate was 78%.

Finally, there is a tendency for individuals to underreport items they consider to cause them to be viewed as deviant or behaving in a socially undesirable way [45]. It is possible that smoking in front of patients and admitting that one is not prepared to help patients quit smoking might be underreported because the physicians are aware of the adverse health consequences that may result from their behavior. Underreporting may also result if smoking is perceived to be socially unacceptable. Whether underreporting occurred and, if so, the extent of underreporting is unknown.

## Conclusion

A high prevalence of smoking was identified among physicians in Yerevan, Armenia. Female healthcare workers have a much higher smoking prevalence than in the overall female population. A high percentage of smokers have smoked in the presence of their patients. Level of perceived preparedness for assisting patients to quit smoking was positively associated with knowledge about known health risks associated with smoking. Only 35% felt well prepared to assist patients to quit smoking. Physicians who smoke are less likely to ask their patients about their smoking behavior or believe their example is likely to influence their patients. These results indicate a need to educate physicians in Armenia of their potential for influencing patients to not start or quit smoking.

## Completing interests

The author(s) declare that they have no competing interests.

## Authors' contributions

Paul C Perrin, MPH, and Ray M Merrill, PhD, MPH, conceptualized and designed the study and participated in the analyses. Paul C Perrin and Gordon B Lindsay participated in collecting the data. All authors participated in drafting and revising the manuscript.

## Pre-publication history

The pre-publication history for this paper can be accessed here:



## Figures and Tables

**Table 1:** Frequency distributions of selected demographic and lifestyle variables for 240 physicians in Yerevan, Armenia, 2004

**Variables**	**No.**	**%**
Age		
20–29	33	14.2
30–39	56	24.1
40–49	55	23.7
50–59	73	31.5
60+	15	6.5
Sex		
Men	105	42.1
Women	133	55.9
Smoking behavior*		
Never smoker	132	55.5
Former smoker	25	10.5
Occasional smoker	13	5.5
Regular smoker	68	28.6
Smoking policy in facility		
None	54	28.0
Special smoking rooms	46	23.8
Completely forbidden to smoke	93	48.2
Is smoking policy enforced?		
Yes, always	94	48.5
Yes, sometimes	42	21.7
No	40	20.6
Don't know	18	9.3
State of preparedness to assist patients to quit smoking		
Well prepared	78	35.3
Somewhat prepared	124	56.1
Unprepared	19	8.6

*Respondents were not provided definitions for the terms regular smoker, occasional smoker, or former smoker. Each respondent defined these terms individually.

**Table 2:** Distribution of 240 physicians by smoking status and sex in Yerevan, Armenia, 2004

**Smoking Status**^†^	**Men**		**Women**	
	**No.**	**%**	**No.**	**%**
Never smoker	26	25.2	105	78.9
Former smoker	20	19.4	5	3.8
Occasional smoker	7	6.8	6	4.5
Regular smoker	50	48.5	17	12.8

*χ^2^(3) = 77.7; *p *< 0.0001.

^†^Respondents were not provided definitions for the terms regular smoker, occasional smoker, or former smoker. Each respondent defined these terms individually.

**Table 3:** Description of current smokers according to sex among 240 physicians in Yerevan, Armenia, 2004

**Variables**	**Men**		**Women**	
	**No.**	**%**	**No.**	**%**
Age started smoking (yrs.)*				
< 20	25	47.2	3	14.3
20–24	19	35.8	4	19.1
25–29	4	7.6	7	33.3
30+	5	9.4	7	33.3
Have you ever quit for at least a week?				
Yes	37	68.5	15	71.4
No	17	31.5	6	28.6
Have you ever smoked in front of patients?*				
Yes	30	52.7	3	13.0
No	26	47.3	20	87.0
Do you regularly smoke in front of patients?				
Yes	1	1.9	1	4.4
No	52	98.1	22	95.6
How ready are you to quit?				
Not ready within the next 6 months	15	42.9	3	21.4
Considering quitting within the next 6 months	12	34.3	5	35.7
Ready to quit now	8	22.9	6	42.9

*χ^2 ^*p*-value < 0.05.

**Table 4:** Level of agreement with selected smoking-related statements for 240 physicians in Yerevan, Armenia, 2004

**Item**	**No.**	**Percent**		
		**Agree**	**Unsure**	**Disagree**
Health professionals should routinely advise patients who smoke to avoid smoking around children.	210	95.7	2.4	1.9
Health professionals should routinely advise their smoking patients to quit smoking.	212	91.0	5.2	3.8
Health professionals should regularly ask their patients about their smoking behaviors.	213	86.9	6.1	7.0
Health professionals should set a good example by not smoking.	209	83.7	8.6	7.7
Health professionals are an example for their patients and the public.	211	75.8	13.3	10.9
Health professionals should speak to lay groups about smoking.	212	75.5	13.2	11.3
Chances of a patient quitting increase when a doctor tells them to.	209	71.3	22.5	6.2
Health professionals should get specific training on cessation techniques.	212	70.2	16.6	13.2
Health professionals who smoke are less likely to advise people to stop smoking.	211	64.9	14.2	20.9

**Table 5:** Odds of being a current smoker according to level of agreement with selected statements for 240 physicians in Yerevan, Armenia, 2004

**Statements**	**Total Respondents**	**% who are current smokers**	Odds Ratio*	95% Confidence Interval*
Health professionals should regularly ask their patients about their smoking behaviors.				
Agree	185	31.2	1.0	(Referent)
Neutral	13	38.5	0.9	0.2, 3.2
Disagree	15	66.7	3.8	1.1, 13.1
Health professionals should set a good example by not smoking.				
Agree	175	28.3	1.0	(Referent)
Neutral	18	61.1	3.3	1.1–10.1
Disagree	16	68.8	5.8	1.7–19.9
Health professionals are an example for their patients and the public.				
Agree	160	27.2	1.0	(Referent)
Neutral	28	50.0	3.2	1.2–8.1
Disagree	23	60.9	3.7	1.4–9.9
Health professionals should get specific training on cessation techniques.				
Agree	149	29.1	1.0	(Referent)
Neutral	35	41.2	1.2	0.5–2.9
Disagree	28	53.6	2.6	1.1–6.5
Health professionals who smoke are less likely to advise people to stop smoking.				
Agree	137	28.9	1.0	(Referent)
Neutral	30	46.7	2.6	1.1–6.5
Disagree	44	43.2	2.1	0.9–4.7

*Adjusted for age and sex.

**Table 6:** Association between feelings of preparedness to counsel patients about smoking and agreement with selected statements about smoking for 240 physicians in Yerevan, Armenia, 2004

	**No.**	**% Well Prepared**	**% Somewhat Prepared**	**% Unprepared**
Smoking is harmful to your health.*				
Agree	188	34.6	58.5	6.9
Unsure/Disagree	12	16.7	50.0	33.3
Neonatal death is associated with passive smoking.*				
Agree	68	52.9	44.2	2.9
Unsure/Disagree	131	24.4	64.9	10.7
Maternal smoking during pregnancy increases the risk of SIDS.*				
Agree	146	38.4	54.8	6.8
Unsure/Disagree	52	19.2	67.3	13.5
Passive smoking increases the risk of lung disease in nonsmoking adults.				
Agree	163	35.6	57.1	7.3
Unsure/Disagree	38	26.2	60.6	13.2
Passive smoking increases the risk of lung disease in nonsmoking adults.*				
Agree	145	38.6	54.5	6.9
Unsure/Disagree	56	21.4	66.1	12.5
Passive smoking increases the risk of lower respiratory tract illnesses, such as pneumonia, in exposed children.				
Agree	141	35.5	58.2	6.4
Unsure/Disagree	59	27.1	59.3	13.6

*χ^2 ^*p*-value < 0.05.

## References

[B1] Rodgers A, Ezzati M, Vander Hoorn S, Lopez AD, Lin RB, Murray CJ (2004). Distribution of Major Health Risks: Findings from the Global Burden of Disease Study. PLoS Med.

[B2] Ezzati M, Lopez AD, Rodgers A, Vander Hoorn S, Murray CJ, Comparative Risk Assessment Collaborating Group (2002). Selected major risk factors and global and regional burden of disease. Lancet.

[B3] Centers for Disease Control and Prevention (CDC) (1999). Best Practices for Comprehensive Tobacco Control Programs.

[B4] Centers for Disease Control and Prevention (CDC) (2005). Smoking Prevalence among US Adults.

[B5] Mackay J, Eriksen M (2002). The Tobacco Atlas.

[B6] Gyurjyan G, Bazarchyan A (2005). Report on the results of the national survey on the drug, alcohol and smoking prevalence among the general population of Armenia.

[B7] World Bank Economics of Tobacco for Armenia.

[B8] National Statistical Service of the Republic of Armenia (NSS) (2004). Statistical Yearbook of Armenia Public Health.

[B9] Peto R, Lopez AD, Boreham J, Thun M, Heath C, Doll R (1996). Mortality from smoking worldwide. Br Med Bull.

[B10] Peto R, Lopez AD, Boreham J, Thun M, Heath C (1992). Mortality from tobacco in developed countries: indirect estimation from national vital statistics. Lancet.

[B11] Foote JA, Harris RB, Gilles ME, Ahner H, Roice D, Becksted T, Messinger T, Bunch R, Bilant K (1996). Physician advice and tobacco use: a survey of 1st-year college students. J Am Coll Health.

[B12] Kunze M (1989). Role of the physician as opinion leader in tobacco control. Chest.

[B13] Duttenhaver JR (1986). Tobacco and health: the role of the physician. J Med Assoc Ga.

[B14] Bialous SA, Sarna L (2004). Sparing a few minutes for tobacco cessation: if only half of all nurses helped one patient per month quit smoking, more than 12 million smokers would overcome their addictions every year. Am J Nurs.

[B15] World no tobacco day 2005 (2005). Health professionals and tobacco control A briefing file for the WHO European Region.

[B16] Tessier JF, Thomas D, Nejjari C, Belougne D, Freour P (1995). Attitudes and opinions of French cardiologists towards smoking. Eur J Epidemiol.

[B17] King G (1997). Attitudes and practices of African-American physicians toward smoking interventions: an earlier study. J Assoc Acad Minor Phys.

[B18] Ndiaye M, Ndir M, Quantin X, Demoly P, Godard P, Bousquet J (2003). [Smoking habits, attitudes and knowledges of medical students of Medicine, Pharmacy and Odonto-Stomatology's Faculty of Dakar, Senegal] [Article in French]. Rev Mal Respir.

[B19] Josseran L, King G, Velter A, Dressen C, Grizeau D (2000). Smoking behavior and opinions of French general practitioners. J Natl Med Assoc.

[B20] Hodgetts G, Broers T, Godwin M (2004). Smoking behaviour, knowledge and attitudes among Family Medicine and nurses in Bosnia and Herzegovina. BMC Fam Pract.

[B21] Pizzo AM, Chellini E, Grazzini G, Cardone A, Badellino F (2003). Italian general practitioners and smoking cessation strategies. Tumori.

[B22] Barengo NC, Sandstrom PH, Jormanainen VJ, Myllykangas MT (2004). Changes in smoking prevalence among Finnish physicians 1990–2001. Eur J Public Health.

[B23] Power B, Neilson S, Perry IJ (2004). Perception of the risks of smoking in the general population and among general practitioners in Ireland. Ir J Med Sci.

[B24] Parna K, Rahu K, Barengo NC, Rahu M, Sandstrom PH, Jormanainen VJ, Myllykangas MT (2005). Comparison of knowledge, attitudes and behaviour regarding smoking among Estonian and Finnish physicians. Soz Praventivmed.

[B25] Josseran L, King G, Guilbert P, Davis J, Brucker G (2005). Smoking by French general practitioners: behaviour, attitudes and practice. Eur J Public Health.

[B26] Soto Mas FG, Papenfuss RL, Jacobson HE, Hsu CE, Urrutia-Rojas X, Kane WM (2005). Hispanic physicians' tobacco intervention practices: a cross-sectional survey study. BMC Public Health.

[B27] National Statistical Service of the Republic of Armenia (NSS) (2001). Tobacco Consumption in the Republic of Armenia.

[B28] Crofton J, Simpson D (2002). Tobacco A Global Threat.

[B29] Satterhwaite FE (1946). An approximate distribution of the estimates of variance components. Biometrics Bulletin.

[B30] Hosmer DW, Lemeshow S (2000). Applied logistic regression.

[B31] Lancaster T, Stead L, Silagy C, Sowden A (2000). Effectiveness of interventions to help people stop smoking: findings from the Cochrane Library. BMJ.

[B32] Gorin SS, Heck JE (2004). Meta-analysis of the efficacy of tobacco counseling by health care providers. Cancer Epidemiol Biomarkers Prev.

[B33] Morbidity and Mortality Weekly Report (MMWR) (1993). Physician and other health-care professional counseling of smokers to quit – United States, 1991. MMWR.

[B34] Bal DG, Lloyd JC, Manley MW (1995). The role of the primary care physician in tobacco use prevention and cessation. CA Cancer J Clin.

[B35] Jonathan M. Samet and Soon-Young Yoon, World health Organization (2001). Women and the tobacco epidemic Challenges for the 21st century.

[B36] Bortz WM (1992). Health behavior and experiences of physicians. Results of Alto medial Clinic physicians. West J Med.

[B37] Morbidity and Mortality Weekly Report (MMWR) (1990). Current trends smoking-related mortality decline among physicians – Rhode Island. MMWR.

[B38] Enstrom JE (1983). Trends in mortality among California physicians after giving up smoking: 1950–79. Br Med J (Clin Res Ed).

[B39] Nelson DE, Giovino GA, Emont SL, Brackbill R, Cameron LL, Peddicord J, Mowery PD (1994). Trends in cigarette smoking among US physicians and nurses. JAMA.

[B40] Frank E, Brogan DJ, Mokdad AH, Simoes EJ, Kahn HS, Greenberg RS (1998). Health-related behaviors of women physicians vs other women in the United States. Arch Intern Med.

[B41] Frank E (2004). Physician health and patient care. JAMA.

[B42] Cummings KM, Giovino G, Sciandra R, Koenigsberg M, Emont SL (1987). Physician advice to quit smoking: who gets it and who doesn't. Am J Prev Med.

[B43] Frank E, Rothenberg R, Lewis C, Belodoff BF (2000). Correlates of physicians' prevention-related practices. Findings from the Women Physicians' Health Study. Arc Fam Med.

[B44] Joint Committee on Smoking and Health (JCSH) (1995). Smoking and health: a physician's responsibility. A statement of the Joint Committee on Smoking and Health. American College of Chest Physicians, American Thoracic Society, Asia Pacific Society of Respirology, Canadian Thoracic Society, European Respiratory Society, International Union Against Tuberculosis and Lung Disease. Monaldi Arch Chest Dis.

[B45] Aday LA (1996). Designing and Conducting Health Surveys.

